# Rapid detection of single bacteria in unprocessed blood using Integrated Comprehensive Droplet Digital Detection

**DOI:** 10.1038/ncomms6427

**Published:** 2014-11-13

**Authors:** Dong-Ku Kang, M. Monsur Ali, Kaixiang Zhang, Susan S. Huang, Ellena Peterson, Michelle A. Digman, Enrico Gratton, Weian Zhao

**Affiliations:** 1 Department of Pharmaceutical Sciences, University of California–Irvine, 845 Health Sciences Road, Irvine, California 92697, USA; 2 Sue and Bill Gross Stem Cell Research Center, University of California–Irvine, 845 Health Sciences Road, Irvine, California 92697, USA; 3 Chao Family Comprehensive Cancer Center, University of California–Irvine, 845 Health Sciences Road, Irvine, California 92697, USA; 4 Edwards Lifesciences Center for Advanced Cardiovascular Technology, University of California–Irvine, 845 Health Sciences Road, Irvine, California 92697, USA; 5 Department of Biomedical Engineering, University of California–Irvine, 845 Health Sciences Road, Irvine, California 92697, USA; 6 Department of Chemistry, Beijing Key Laboratory for Analytical Methods and Instrumentation, Tsinghua University, Beijing 100084, China; 7 Division of Infectious Diseases and Health Policy Research Institute, School of Medicine, University of California–Irvine, Irvine, California 92697, USA; 8 Department of Pathology and Laboratory Medicine, University of California, Irvine, California 92697, USA; 9 Laboratory for Fluorescence Dynamics, University of California, Irvine, California 92697, USA; 10 Centre for Bioactive Discovery in Health and Ageing, School of Science and Technology, University of New England, Armidale, New South Wales 2351, Australia

## Abstract

Blood stream infection or sepsis is a major health problem worldwide, with extremely high mortality, which is partly due to the inability to rapidly detect and identify bacteria in the early stages of infection. Here we present a new technology termed ‘Integrated Comprehensive Droplet Digital Detection’ (IC 3D) that can selectively detect bacteria directly from milliliters of diluted blood at single-cell sensitivity in a one-step, culture- and amplification-free process within 1.5–4 h. The IC 3D integrates real-time, DNAzyme-based sensors, droplet microencapsulation and a high-throughput 3D particle counter system. Using *Escherichia coli* as a target, we demonstrate that the IC 3D can provide absolute quantification of both stock and clinical isolates of *E. coli* in spiked blood within a broad range of extremely low concentration from 1 to 10,000 bacteria per ml with exceptional robustness and limit of detection in the single digit regime.

## Abstract (web summary)

Early detection of blood stream infections is essential for providing effective treatments. Here the authors present a system integrating DNAzyme sensors, droplet microfluidics and a high-throughput 3D particle counter that can detect specific, single bacterial cells in blood within a few hours.

## Graphical abstract

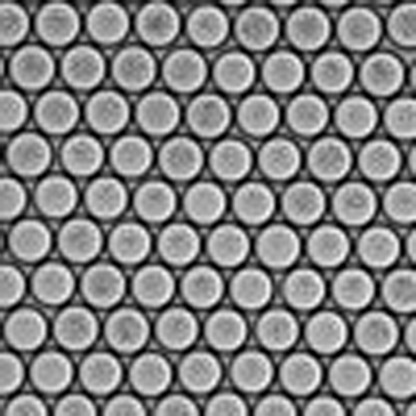

Blood stream infections (BSIs) are a major cause of morbidity and mortality. Sepsis resulting from a BSI annually affects over 18 million people worldwide and 700,000 in the United States, with a mortality rate of 30–40% (refs 1, 2) Sepsis and other aggressive bacterial infections associated with BSIs are often times managed within intensive care units with associated high costs, which impose significant health-care, economic and social burdens. For instance, each septic patient in the United States incurs costs of approximately $25,000 during hospitalization, corresponding to $17 billion annually^1^,^2^. The extremely high mortality of blood infections is due, in part, to the inability to rapidly detect, identify and thus treat patients with appropriate antibiotics in the early stages of infection^1^,^2^,^3^. The initial treatment with empirical broad-spectrum antibiotics not only is inadequate but also encourages antibiotic resistance^4^,^5^. It is widely recognized that effective detection and monitoring of patients to diagnose a BSI at an early stage have a profound effect on survival rates^1^,^2^,^3^. However, the present gold standard, bacterial blood cultures coupled with susceptibility testing for drug resistance, requires days to obtain a result. Recent amplification-based molecular diagnosis methods including PCR can reduce the assay time to hours but are often not sensitive enough to detect bacteria that occur at low concentrations in blood (≪1–100 colony-forming unit (CFU) per ml) as is commonly found in adults with BSI and therefore often still require a culture-enrichment step^6^,^7^. Moreover, these conventional methods typically suffer from poor specificity and high background signal because a target bacteria is surrounded by billions of non-target species (for example, red blood cells) in the blood sample. More recent nano- and micro-systems including droplet microfluidics (for example, digital PCR)^8^,^9^,^10^,^11^,^12^,^13^,^14^,^15^,^16^,^17^,^18^ can improve detection sensitivity and selectivity but typically are limited to microlitre sample volume, which cannot handle the required clinical sample volume (millilitres) and throughput. Inevitably, the existing methods typically require expensive equipment and lengthy, complex sample processing (for example, cell lysis, nucleic acid extraction, centrifugation, magnetic separation, washing and signal amplification) for target purification and enrichment, which not only results in significant loss of rare target organisms, and therefore contributes to a high false-negative rate, but also limit their widespread use especially in a point-of-care setting (for example, in an ambulance)^6^.

We present herein a platform technology called Integrated Comprehensive Droplet Digital Detection (IC 3D) that is able to selectively detect bacteria from millilitres of unprocessed (although diluted) blood at single-cell sensitivity in a one-step, and culture- and amplification-free reaction within 1.5–4 h.

## Results

### Overview of IC 3D

**Figure 1:**
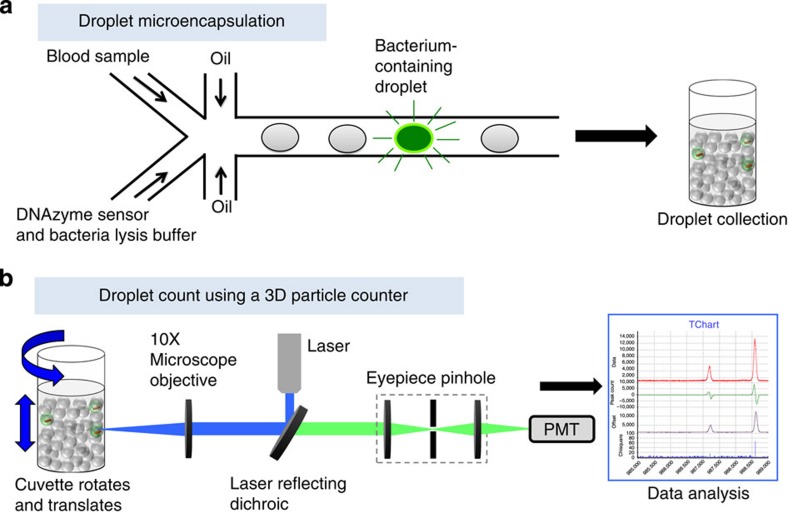
Schematic description of the IC 3D technology. (**a**) Blood samples and DNAzyme sensors are mixed and then encapsulated in 100 s of millions of micrometre-sized droplets. DNAzyme sensors produce an instantaneous signal in the droplets that contain the bacterium. (**b**) In the IC 3D, droplets are collected and analysed using our high-throughput 3D particle counter that permits accurate detection of single-fluorescent droplets in a several millilitre pool of non-fluorescent droplets within minutes.

Our IC 3D system integrates real-time DNAzyme sensor technology, droplet microfluidics and a high-throughput 3D particle counter system (Fig. 1). DNAzyme sensors used here are short catalytic oligonucleotides that are identified by *in vitro* evolution to specifically react with the lysates of target bacteria, leading to a rapid fluorescence signal^19^. Specifically, blood samples are mixed with the DNAzyme sensor solution including bacteria lysis buffer within a microfluidic channel, which is then immediately encapsulated into 100 s of millions of individual picolitre droplets. The confinement of bacteria in droplets that serve as ‘microreactors’ significantly increases (i) the concentration of released target molecules in the droplets that contain bacteria such that single bacteria can be detected by the DNAzyme sensors in a rapid manner and (ii) target/background ratio to minimize interference from nonspecific binding and noise. In the IC 3D system, droplets are collected following generation into a vial and analysed using a high-throughput 3D particle counting system. The 3D particle counter was recently developed by Gratton and colleagues and can robustly and accurately detect single-fluorescent particles from millilitre volumes within minutes^20^,^21^. In the IC 3D system, the compartmentalized, target-specific reaction mediated by DNAzyme sensors is critical to ‘light up’ the droplets that contain target bacteria so that they can be detected by the 3D particle counter with exceptionally high reliability and clinically relevant throughput.

### DNAzyme sensors selectively detect target bacteria in bulk

DNAzymes (also called ‘DNA enzymes’ or ‘deoxyribozymes’) are synthetic single-stranded DNA oligonucleotides with catalytic activities^22^,^23^. These catalytic DNA molecules are generated *in vitro* from a vast random library using a combinatorial approach called *in vitro* evolution^24^,^25^. One of the most established DNAzymes is a RNA-cleaving DNA motif that can cleave a DNA–RNA chimeric substrate at a single-ribonucleotide junction^22^,^26^. Ali and colleagues have recently harnessed this unique property to generate bacteria-specific DNAzyme sensors through *in vitro* evolution of a vast DNA library against crude extracellular matrix (CEM) components of target bacteria as complex targets^19^. A later study indicated that the cell lysate produces much higher fluorescence signal compared with the CEM by the DNAzyme sensors, suggesting that the cells contain higher amount of targets than CEM^27^. In our study, therefore, we decided to lyse the cells to facilitate detection and first tested a series of detergents and enzymes to optimize the cell lysis condition and identified that lysozyme most efficiently lyses bacteria without interfering with droplet generation and stability or DNAzyme sensor function (see Methods section for details).

**Figure 2:**
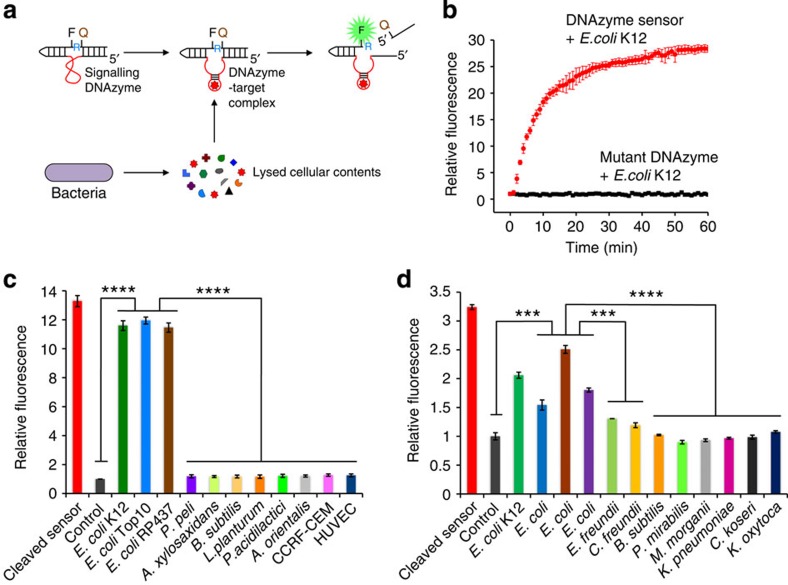
Real-time DNAzyme sensors selectively and rapidly detect target *E. coli* lysates in bulk. (**a**) Proposed mechanism of how the DNAzyme sensor generates a fluorescent signal upon interaction with the target. The target(s) produced by the bacterium binds to the inactive DNAzyme sequence (red), which undergoes a conformational change to activate the DNAzyme. The activated DNAzyme catalyses the cleavage of the fluorogenic substrate at the ribonucleotide junction (R), leading to the separation of the fluorophore (F) and the quencher (Q) to produce a high-fluorescence signal. (**b**) DNAzyme sensor produces real-time fluorescence signal in the presence of the target *E. coli* K12 lysates. By contrast, a mutated DNAzyme sequence is inactive. Lysates from 10,000 bacteria and 50 nM DNAzyme were mixed in a 50-μl final volume in HEPES buffer and signal was recorded using a fluorescence plate reader. Results are shown as mean±s.e.m (*n*=3). (**c**) DNAzyme sensor specifically detects *E. coli* strains but not non-target bacteria or mammalian cell human T-cell lymphoblast CCRF-CEM and human umbilical vein endothelial cells (HUVECs). Lysates from 10,000 cells and 50 nM DNAzyme were mixed in a 50-μl final volume in HEPES buffer and incubated for 30 min. DNAzyme reaction products were analysed by polyacrylamide gel electrophoresis. The percentage cleavage for each reaction was derived, normalized against DNAzyme alone control and presented as ‘Relative fluorescence’. (**d**) DNAzyme sensors can selectively detect clinical *E. coli* isolates. Bacteria (1,000 CFU) isolated from 11 different patient samples were incubated with 100 nM DNAzyme and 1 mg ml^−1^ lysozyme in 10% of blood for 30 min. Fluorescence intensity was obtained using a fluorescence plate reader, normally against DNAzyme alone control (con) and presented as ‘Relative fluorescence’. Data are obtained in a single-blind experiment. In **c** and **d**, all experiments were performed in triplicate. Data are represented as mean±s.d., *n*=3, ***P≪0.001, ****P≪0.0001, two-tailed Student’s *t*-test.

We sought to use these rapid, fluorogenic DNAzyme sensors in our IC 3D system. As shown in Fig. 2a, the sensor contains a DNAzyme domain that is ligated with the DNA–RNA chimeric substrate where the ribonucleotide cleavage site is flanked by a fluorophore and a quencher. This ‘inactive’ state has a minimal fluorescence signal because of the close proximity of the fluorophore and the quencher. In the presence of target bacteria, *E. coli* used herein as a model system, DNAzymes will bind to target molecules produced by bacteria and cleave the substrate. The cleavage event (Supplementary Fig. 1) frees the fluorophore from its quencher, thereby generating a high fluorescence signal (Fig. 2b). Moreover, the DNAzyme sensor is able to distinguish target *E. coli* from control bacteria or mammalian cells with high selectivity (Fig. 2c). We further demonstrate that the DNAzyme sensors previously isolated using stock isolates of *E. coli*^19^ can robustly and selectively detect clinical *E. coli* isolates that were spiked and then lysed in blood (Fig. 2d). It is interesting to note that although the DNAzyme sensor can detect all clinical *E. coli* samples, the fluorescence intensity varies between samples, which might reflect the potential molecular heterogeneity between different *E. coli* strains. Furthermore, fluorescence intensity appeared to be slightly increased in the *C. freundii* groups comparing to the blood alone control but it was statistically significantly less than the *E. coli* groups (*P*≪0.001, two-tailed Student’s *t*-test) and was not statistically different than other clinical control isolates (*P*=0.85, two-tailed Student’s *t*-test). Nonetheless, this points out the need to rigorously validate the sensor specificity using larger number of patient samples in the future. This also suggests that by including appropriate positive and negative selection targets in the *in vitro* evolution process, it is feasible to generate DNAzyme sensors that can distinguish different strains of the same bacterium species, which will need to be validated in the future work. As our goal is to develop a ‘mix-and-read’ assay that uses whole blood with no or minimal sample processing (for example, dilution), we further tested the sensor performance in blood and found that our Fluorescein/Dabcyl-modified DNAzyme sensors produced a sufficiently high fluorescence signal in response to *E. coli* spiked in blood that was diluted by sensor solution to various volume ratios (Supplementary Fig. 2a) with a 10% final blood concentration determined to be optimal and therefore used in subsequent droplet experiments (below). We hypothesize that optimization of dye pairs, especially using near infrared dyes that are less interfered with by blood autofluorescence in the future, can further improve sensor performance (for example, signal/noise ratio) in blood. We further demonstrated that the DNAzyme sensors exhibited sufficient stability in blood within the time frame (≪1.5–4 h), we target for future clinical use (Supplementary Fig. 2b). If necessary, the use of well-established chemically modified nucleotides (for example, phosphorothioates) in DNAzymes or RNase inhibitor in the assay buffer can further increase sensor stability in blood.

### DNAzyme sensors rapidly detect single bacteria in droplets

We next integrated DNAzyme sensors with the droplet microfluidics system to test our hypothesis that the confinement of bacteria in droplets can significantly increase the sensitivity (that is, single-cell) and shorten the detection time. The ‘droplet microfluidics’ system enables the generation and manipulation of monodisperse, picolitre-sized (typically 5 to 100 μm in diameter) liquid droplets in an immiscible carrier oil fluid (that is, water-in-oil emulsion)^28^,^29^,^30^,^31^,^32^,^33^. Droplet microfluidics is an emerging platform for ultra-sensitive biological detection and analysis. In particular, the pioneering work from the Weitz and Griffiths groups and from companies including Bio-Rad and Raindance have demonstrated a range of droplet-based ‘digital’ assays for nucleic acids (for example, digital PCR), cells and organisms^10^,^31^,^34^. However, the traditional droplet system is limited by its low throughput in droplet analysis and therefore not amenable for analysis of large volume samples (see the ‘Rapid detection of single bacteria in clinical blood samples’ section)^31^,^34^.

**Figure 3:**
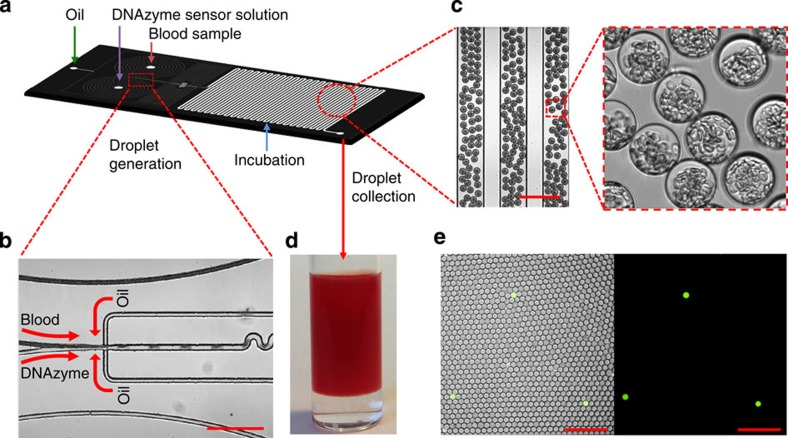
Workflow of microencapsulation. (**a**) Layout of the droplet-based microfluidic device. Devices were designed with three inlets; one for oil and the other two for blood samples and DNAzyme/bacterial lysis buffer. (**b**,**c**) Representative microscopy images showing uniform 30 μm droplets containing 10% blood and sensor solution are being generated using flow focusing. Scale bar, 200 μm. In **c**, blood contents especially red blood cells are clearly visible in droplets. (**d**) Droplets collected in the cuvette used for 3D particle counter experiments. (**e**) Representative fluorescence microscope images demonstrate DNAzyme sensors (250 nM) light up the droplets that contain single *E. coli* K12 in 10% blood after 3-h reaction. Left panel: overlay of fluorescence and brightfield. Right panel: fluorescence. Scale bar, 200 μm.

In our study, droplet microfluidics were fabricated using standard soft lithography and operated following previously established procedures (see ‘Fabrication of droplet-based microfluidic device’ in Methods)^35^. As illustrated in Fig. 3a, the poly(dimethylsiloxane) (PDMS) chip has one oil inlet and two aqueous inlets (one for bacteria containing buffer or blood with the other one for the DNAzyme sensor mixed with bacterial lysis reagent (lysozyme)). Uniform picolitre-sized droplets were generated using standard syringe pumps at a rate of approximately 2,000 Hz by flow focusing of the resulting stream with HFE-7500-fluorinated oil containing 1.8% (w/w) perfluorinated polyethers with polyethyleneglycol surfactant (Fig. 3b and Supplementary Movie). We generated droplets with different sizes ranging from 5 to 50 μm in diameter simply by tuning the microfluidic channel size and flow rate. Figure 3c shows a representative image of 30 μm droplets that contain 10% blood. We found that these droplets can be stably stored without leaking or merging for months at room temperature even when closely packed in a vial. High-throughput droplet generation can be achieved using a multi-layer microfluidic device that contains multiple, parallel droplet-generating structures (see ‘Fabrication of droplet-based microfluidic device’ in Methods and Supplementary Fig. 3 for an eight-channel device). To achieve clinically useful throughput, we are currently developing a device that contains 256 droplet-generating channels, which is able to convert 1 ml blood to 25 μm droplets containing 10% blood at a generation rate of 2,000 Hz per channel in ≪40 min. In addition, the use of larger droplet and smaller blood dilution factor can further significantly reduce the droplet generation time.

As in adult BSI, bacteria are in low numbers in patient’s blood (typically 1–100 CFU ml^−1^), when encapsulated in picolitre droplets, each droplet will contain one or no bacterium. Therefore, it is critical to test if our system is able to detect a single bacterium in a droplet. Specifically, bacteria were statistically diluted to achieve a range of concentrations from 10 to 10^7^ ml^−1^ spiked in HEPES buffer or blood and compartmentalized in droplets. In some experiments, bacteria were stained with Syto17 (red), which allows us to co-localize the bacterium with the DNAzyme sensor signal (green) in the same droplet to determine false-positive and -negative rates. In this section, fluorescent droplets were imaged by conventional fluorescent (or confocal) microscopy or counted on chip in a 1D microfluidic channel at a throughput of ~200 droplets per s using a custom-built confocal microscope equipped with APD detectors (see ‘1D on-chip detection system’ in Supplementary Methods). We first demonstrate that, in buffer, the DNAzyme sensor system is able to detect single-target *E. coli* K12 that is lysed in a droplet (5 μm in diameter) within 8 min (Supplementary Fig. 4). This pilot experiment tested our hypothesis that the confinement of bacteria in droplets enables single-cell sensitivity and reduced detection time. For detection of bacteria in blood, we needed to optimize droplet size: although smaller droplet sizes lead to higher target concentrations from single cells (which would increase the signal/background ratio and decrease the detection time), it is technically challenging to encapsulate blood contents including red and white blood cells into too small-sized droplets. We determined that droplets 25 μm in diameter are optimal for this purpose and therefore used for subsequent blood droplet experiments. Using fluorescent microscopy (Fig. 3e) or 1D on-chip droplet counting system (Supplementary Fig. 5), our system is able to selectively detect single-target *E. coli* K12 in 10% blood in droplets. Furthermore, by co-localizing with the Syto17 signal, we observe that our encapsulated DNAzyme sensor system possesses zero false-positive rate and minimal false-negative rate (~0.5%) from ~70,000 droplet counts in triplicate experiments, which we performed using *E. coli* K12 as positive target and sensor alone or control bacteria as negative controls (Supplementary Fig. 5). Finally, although the DNAzyme sensor signal generation in a 25-μm blood droplet is not as rapid as that in a 5-μm buffer droplet, a measurable fluorescence signal can be observed within 3 h in response to a single bacterium in blood (Fig. 3e and Supplementary Fig. 5; see below for further DNAzyme kinetics study).

### Rapid detection of single bacteria in clinical blood samples

We have demonstrated above that the encapsulated DNAzyme sensor system can detect target bacteria in a rapid manner with single-cell sensitivity in the droplet. However, the 1D on-chip droplet counting system (which is also used in the droplet digital PCR system) and other particle counting systems including flow cytometry suffer from low throughput: they typically operate at 1,000 s particles per s and are only able to analyse a total of 100,000 s to 1 million droplets (or a total sample volume of ~tens of microlitre)^31^,^34^. Therefore, the existing droplet detection systems inevitably require sample preparation to purify and enrich targets and reduce sample volume before droplet encapsulation. In our system, however, we want to rapidly analyse unprocessed patient blood with a clinical sample volume of typically millilitres that translates up to billions of droplets. To effectively analyse these many droplets in a short period of time and detect single fluorescent, bacteria-containing droplets among millions of empty ones, in the IC 3D system, we integrated a 3D particle counter^21^, as we described earlier, that can detect fluorescent particles from millilitre volumes at single-particle sensitivity within minutes. Briefly, as shown in Fig. 1b and Supplementary Fig. 6, the custom-built prototype apparatus consists of a small, portable microscope that has a horizontal geometry and a mechanical part that holds a cylindrical cuvette with a diameter of 1 cm. Two motors provide rotational (ranging from 10 to 1,100 r.p.m.) and vertical up-and-down motion (ranging from 1 to 15 mm s^−1^) of the cuvette. The excitation light generated by a diode laser (469 nm) is focused at the volume of observation that is typically positioned relatively close to the inside wall of the cuvette. The emission from the sample is collected by the same objective, transmitted through the set of dichroic filters, focused by a lens into a pinhole and then collimated by a second lens to the photomultiplier tube (PMT). The optics of the microscope is designed to measure a relatively large volume (100 pl) in about 0.01 ms. The rotation of the tube in a spiral motion for about 100 s allows us to effectively explore about 1 ml of the tube. We emphasize that using this optical setup, we are penetrating only 150 μm into the sample. Therefore, strongly scattering samples such as whole blood (even before dilution) that have a transmittance at 500 nm of about 10% for a 250-μm path length can be easily handled.

**Figure 4:**
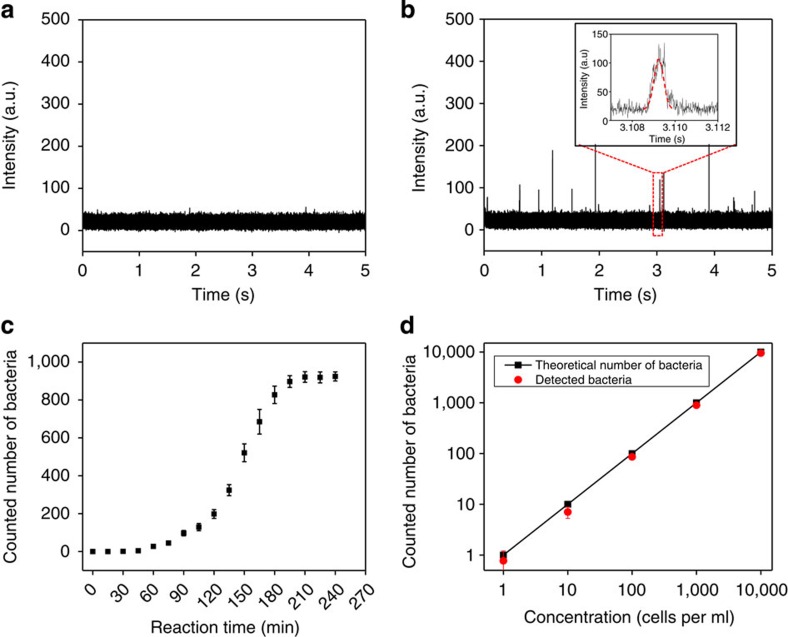
Detection of bacteria in blood samples using the IC 3D system. The generated droplets (25 μm in diameter) containing DNAzyme sensors (250 nM) and 10% blood spiked with bacteria were collected (2 ml) in a cuvette and analysed by a 3D particle counter. (**a**) Donor blood alone (without bacteria) mixed with DNAzyme sensors did not show any signal. (**b**) A representative bacteria sample measurement shows a typical time trace with fluorescence intensity spikes obtained from droplets containing a single *E. coli* K12. The temporal profile is analysed with a pattern-recognition algorithm (inset box) to extract the measurement of the concentration and/or brightness of the droplets in the sample. In this set of experiments, bacteria-spiked blood was incubated with DNAzyme in droplets for 3 h. The bacteria concentration was 1,000 CFU ml^−1^ of droplet solution. (**c**) DNAzyme reaction kinetics for quantitative bacterium detection in blood droplets measured by the 3D particle counter. A total of 1000 bacteria were spiked in this sample. Fluorescent droplets were quantified every 15 min using a 3D particle counter (Supplementary Fig. 7, Supplementary Table 1) and the number of bacteria detected was plotted as *y* axis as function of DNAzyme reaction time. Data are represented as mean±s.d., *n*=3. (**d**) Actual counted cell numbers using the IC 3D (*y* axis) versus a broad range of spiked bacteria concentration (*x* axis: numbers of bacteria per milliliter of collected droplet solution; the raw data are presented in Supplementary Table 2). *Y*=0.95*X*. *R*^2^=0.999. To precisely achieve extremely low bacterial concentration (1–50 cells ml^−1^), bacteria were collected and spiked into blood using a microinjector system before encapsulation. Bacteria-spiked blood was incubated with DNAzyme in droplets for 3 h in this set of experiments. Data are represented as mean±s.d, *n*=3. Note that the error bars for concentrations of 100, 1,000 and 10,000 cells per ml are too small to see.

**Figure 5:**
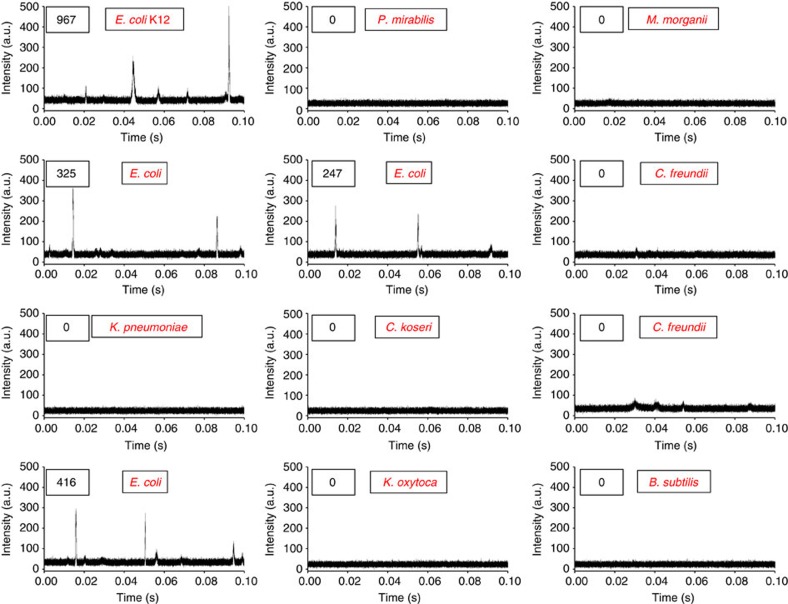
The IC 3D system can selectively detect clinical *E. coli* isolates. Representative 3D particle counter data demonstrate that only target *E. coli* isolate among 11 different bacterial isolates generate typical fluorescence intensity spikes in a single-blind experiment. The total number of counted cells in each sample is shown in the boxes in top left corner. *E. coli* K12-spiked blood was used as a positive control.

We encapsulated bacteria-spiked blood and DNAzyme sensors into droplets as we described previously. Droplets were collected in a cuvette (Fig. 3d) and then analysed by the 3D particle counting system. Using this system, we have demonstrated that fluorescent droplets that contain single target *E. coli* K12 and DNAzyme sensors can be detected at single-droplet sensitivity from a typical 2 ml sample volume within 3 min measurement time (Fig. 4a,b). Our current system typically operates at a throughput of ~100,000 s droplets per s or an effective volume of observation of ~0.1 ml min^−1^. With such a high throughput, the sample volume increase resulting from blood dilution in our experiments becomes less a problem. Figure 4b shows a typical time trace with fluorescence intensity spikes obtained from bacterium-containing droplets. It is important to emphasize that in our IC 3D assay, the detection of a ‘hit’ is defined by a pattern-recognition algorithm (Fig. 4b, inset box and ‘Principle of the pattern recognition algorithm and quantitative criterion for detection’ in Methods) rather than threshold intensity (which is widely used in conventional 1D particle counting systems and typically suffers from higher false-positive/negative rates because the intensity is dependent on many factors including lasers and detectors). Briefly, a fluorescent particle (a droplet in our paper) is detected by the ‘shape’ produced by the passage of the particle in the volume of illumination, which is Gaussian for our instrument. The pattern recognition detects the time of the passage of the particle and the amplitude of the detected pattern. Predetermined using fluorescent droplets that contain already cleaved or reacted DNAzyme sensors (the ‘standard’), our pattern-recognition algorithm can automatically filter the noise and only report true bacterium-containing, fluorescent droplets. Such pattern recognition allows us to achieve exceptionally reliable and accurate detection of a low concentration of fluorescent droplets in large sample volumes, which translates to essentially zero false-positive rate (that is, a ‘hit’ is always a true positive even among hundreds of millions of empty droplets). This is supported the 0 total count for control samples including healthy donor blood samples without bacteria (*n*=5; see Fig. 4a for a representative time trace and Supplementary Table 2 for complete count) or spiked with non-target clinical bacterial isolates (*n*=8; Fig. 5 and Supplementary Table 2).

To determine the minimal DNAzyme reaction time that is required in our IC 3D system to detect bacteria in unprocessed blood, we monitored the signal from a 2-ml droplet solution over time using our 3D particle counter (Fig. 4c, Supplementary Fig. 7 and Supplementary Table 1). We observed that, in as little as 45 min of DNAzyme reaction, the IC 3D test can generate a ‘yes or no’ result, whereas 3.5 h is typically required to provide absolutely quantitative data about the number of cells in the sample. We next demonstrate that our system can provide absolute quantification of target bacteria at a broad range of extremely low concentration from 1 to 10,000 bacteria per ml with single-cell sensitivity and an exceptional limit of detection (LOD) in the single digit regime (Fig. 4d and Supplementary Table 2 for raw data and errors). There is exceptional linear correlation between the detected number of droplets and the actual concentration of targeted bacteria spiked in the blood sample. Regarding the false-negative rate and analytical errors in these positive samples, for concentrations of 10–10,000 cells per ml, we are always able to detect target *E. coli* despite of the analytical errors, that is, report as ‘positive’ in a ‘yes or no’ test, with essentially 0 false-negative rate. For samples of 1 cell per ml, our assay typically detects the bacterium ~77% of the time. Note that that the time of the measurement could be expanded to decrease the errors^20^,^21^. Therefore, the LOD lies in the single digit regime. Finally, to demonstrate the potential clinical applicability, we tested our system using clinical bacterial isolates obtained from positive blood cultures. We found that our IC 3D system can selectively and robustly detect clinical *E. coli* isolates with a performance similar to what we observed for positive control *E. coli* K12 (Fig. 5).

## Discussion

We have developed a new IC 3D system that integrates real-time sensors, droplet microfluidics and a 3D particle counter for rapid, absolute quantification of low abundant target markers in unprocessed (although diluted), large volume biological samples. The IC 3D system simultaneously satisfies numerous important bioanalytical parameters including sensitivity, selectivity, assay time, throughput and robustness, which is a long-standing unmet challenge in biodetection.

In our proof-of-concept system, the real-time and ‘mix-and-read’ DNAzyme sensors were obtained by *in vitro* selection, which can be used to quickly generate specific DNAzymes for, in principle, any complex targets, including slowly growing organisms (for example, *Mycobacterium tuberculosis*). Given that BSIs and sepsis can be caused by several different types of bacteria and fungi, our ongoing effort aims to expand the sensor set through *in vitro* DNAzyme sensor selection to detect the pathogen species that are most commonly recovered from BSIs. In particular, the nonbiased screening using bacteria as a complex target without prior knowledge of any specific target molecules bypasses the tedious process of purifying and identifying target molecules from extremely complex mixtures and permits the rapid development of sensors for new bacterial strains in an unanticipated outbreak. This addresses a major challenge faced by existing techniques including PCR that rely on the detection of pre-identified target genes or other biomarkers given the rapid and complex evolving mechanisms associated with bacteria. Although the identification of specific bacterial biomarkers that bind to DNAzymes to trigger substrate cleavage is neither necessary for our assay to operate nor the focus of this paper, they can be identified by using affinity purification coupled with mass spectrometry, which is part of our ongoing work.

Moreover, the compartmentalization of a single bacterium in a droplet significantly increases the concentration of target molecules, permitting rapid detection and single-cell sensitivity without signal amplification processes such as PCR. Compartmentalization of target-specific reactions is a critical step to ‘light up’ the droplet ‘reactors’ that contain target bacteria so that they can be detected by the 3D particle counting system.

Furthermore, our 3D particle counting system for single-droplet detection in millilitre volumes within minutes bypass many challenges faced by 1D on-chip counting systems and flow cytometry that suffer from limited throughput and high false-positive/negative rates. The exceptional reliability, accuracy and throughput uniquely distinguish the IC 3D from the conventional droplet microfluidic systems (for example, digital PCR). The IC 3D can provide absolute quantification of target bacteria in blood at a broad range of concentrations from 1 to 10,000 bacteria per ml within ~1.5–4 h (droplet generation (≪40 min)+DNAzyme sensor reaction (~45 min for ‘yes or no’ and ~3.5 h for absolute quantification)+3D particle counting (3–10 min)+data processing (5 min)) with single-cell sensitivity and an exceptional LOD in the single digit regime. We summarized the major performance specifications in Table 1 with comparisons to PCR tests (for example, FilmArray) that were approved by the Food and Drug Administration for bacterial detection. Collectively, our IC 3D system significantly shortens assay time allowing BSI to be treated timely and effectively thereby reducing morbidity and mortality. Our ongoing work focuses on (i) validation of the IC 3D using larger number of patient specimens with respect to clinical sensitivity, specificity and assay time with head-to-head comparisons to gold standard assays including blood culture and PCR, and (ii) development of an automated, portable device that permits multiplex and rapid detection of antibiotic-resistant strains.

We believe that the IC 3D system would be relatively inexpensive compared with existing analytical equipment including PCR and flow cytometry. For instance, according to the potential manufacture of this instrument, the predicted cost of all parts of the 3D particle counter is about $1,000 including optics, detection PMT and computer interface (see Supplementary Table 3).

Furthermore, to enable multiplex and parallel detection of multiple pathogens, we plan to develop a device comprised of multiple laser sources and detectors capable of reading at different wavelengths. The multiplex system would permit simultaneous reading of multiple sensors (labelled in different colors) coded for different pathogens. A carousel could also be added to our apparatus to accommodate multiple sample vials for carrying out parallel tests.

Finally, future integration of other sensing methods (for example, enzymatic assays, PCR and isothermal signal amplifications) with droplet microfluidics and a 3D particle counter may potentially serve as a platform for rapid detection and analysis of almost any type of low abundant markers in biological samples including cells (for example, bacteria, circulating tumor cells and stem cells), extracellular vesicles (for example, exosomes), viruses (for example, HIV) and molecular markers (for example, nucleic acids and proteins). In particular, the IC 3D system can potentially integrate fluorogenic substrates^36^ for beta-lactamases and carbapenemases, which would allow the detection of extended spectrum beta-lactamase-producing *Enterobacteriaceae* and carbapenem-resistant *Enterobacteriaceae* that are among the most prevalent antimicrobial-resistant pathogens.

## Methods

### Preparation of the DNAzyme sensor

The DNAzyme construct used in this study consists of a fluorogenic substrate (FS: 5′-ACTCTTCCTAGCF-rA-QGGTTCGATCAAGA-3′ (F-Fluorescein-dT, rA-Riboadenosine, Q-Dabcyl-dT)) and a catalytic sequence (RFD-EC1: 5′-CACGGATCCTGACAAGGATGTGTGCGTTGTCGAGACCTGCGACCGGAACACTACACTGTGTGGGATGGATTTCTTTACAGTTGTGTGCAGCTCCGTCCG-3′)^19^. The fluorogenic substrate and catalytic oligonucleotides were purchased from the Keck Oligonucleotide Synthesis Facilities at Yale University and Integrated DNA Technologies (IDT), respectively. The tert-butyldimethylsilyl group at the 2′-hydroxyl position of riboadenosine in FS was deprotected and purified following previously reported protocol^37^. The FS and RFD-EC1 were covalently joined through template-mediated enzymatic ligation using the template (LT: 5′-CTAGGAAGAGTCGGACGGAGCTG-3′, IDT) following a previously reported protocol^19^. Briefly, 1 nmol of FS was phosphorylated using 10 U of polynucleotide kinase (Thermo Fermentas) in a 50-μl reaction volume for 30 min in the presence of 1 mM ATP and the polynucleotide kinase buffer. The reaction was quenched by heating the mixture at 90 °C for 5 min and then cooling down to room temperature for 20 min. Equal amounts of LT were added to the reaction mixture, heated at 90 °C for 1 min, then cooled to room temperature for 10 min. Then, 20 μl of 10 × T4 DNA ligase buffer (Thermo Fermentas) was added and the total volume was adjusted to 200 μl with ddH_2_O. Next, 20 U of T4 DNA ligase was added and the mixture was incubated at room temperature for 1 h. The ligated DNA products in the mixture were concentrated by ethanol precipitation and purified by 10% denaturing polyacrylamide gel electrophoresis. A mutant DNAzyme, used as a control, was also synthesized (mRFD-EC1: 5′-CACGCTGTACGGATGGAGTCGCGAGCCTGCGACCGGAAATGAAAGATCTTTCGCGTTTTGCTCATGCGATGGATTTTTTACAGTGGGCAGCTCCGTCCG-3′) and ligated to the substrate as described above.

### Fabrication of droplet-based microfluidic device

A schematic of the fluidic chip design and operating mechanism is shown in Fig. 3a. Microchannel architectures were designed using AutoCAD (Autodesk) and transferred to high-resolution photomasks fabricated on transparencies (CAD/Art Services). The microfluidic device has an oil inlet and two aqueous inlets (one for the bacteria sample to be analysed and the other for the DNAzyme sensor and cell lysis reagents) and a flow-focusing structure that forms droplets through a flowing oil phase. Following droplet formation, a short ‘wiggle’ module is incorporated for rapid mixing of droplets by chaotic advection. The microfluidic channels (width: from 10 to 200 μm, depth: 20–50 μm) were fabricated from PDMS using standard soft lithographic techniques^38^. Briefly, PDMS base and curing agent (Sylgard 184; Dow Corning) were mixed in a ratio of 10:1 w/w, degassed, decanted onto SU8-on-Si wafer master (IDB Technologies Ltd) and fully cured overnight in an oven at 65 °C. After thermal curing, the PDMS layer was peeled off from the master, followed by the punching of inlet, interlayer connecting and outlet holes with a 1-mm sized punch (BIOPSY punch, Kay Industries Co.). PDMS layers and glass cover slides were bonded immediately following plasma exposure.

For the high-throughput droplet generation (Supplementary Fig. 3), two PDMS layers were designed and fabricated. The top layer consists of a flow stream splitting structure and sample mixing junctions between the blood and DNAzyme solutions. The bottom layer consists of eight-parallel droplet generation modules that are connected through interlayer connecting holes between mixing junctions (top layer) and the flow-focusing structure (bottom layer). The 1-mm glass microscope slides were bonded immediately after plasma exposure.

### Blood microencapsulation with DNAzyme sensors

Different cell numbers of *E. coli* K12 or clinical isolates were obtained through serial dilution or, at extremely low numbers (1–50), through a microinjection system, and were spiked in 20% fresh donor blood. Bacteria-spiked blood sample was then microencapsulated with DNAzyme sensor and bacterial lysis agent (lysozyme) using a droplet microfluidic device. Unprocessed whole blood can also be directly encapsulated if diluted by the sensor solution to various blood concentrations to reduce the background signal. On-chip dilution is well established^39^,^40^ and can be performed by changing the relative flow rates of two aqueous streams between blood and sensor. The obtained droplets typically contain one or no bacterium, 250 nM DNAzyme sensor and 1 mg ml^−1^ lysozyme in 10% blood.

Specifically, for microencapsulation of blood samples via the droplet microfluidic device, HFE-7500 fluorocarbon oil (Dow Corning) containing 1.8% (w/w) perfluorinated polyethers with polyethyleneglycol surfactant was used as a continuous phase. Aqueous and oil phases were injected into the microfluidic device via pressure equalization tubes (Smith Medical). In all microfluidic experiments, PHD 2000 syringe pumps (Harvard Apparatus) were used to inject liquids at flow rates ranging from 0.5 to 5 μl min^−1^. Briefly, the 20% blood samples were prepared by diluting with phosphate-buffered saline (137 mM NaCl, 2.7 mM KCl, 10 mM Na_2_HPO_4_, 2 mM KH_2_PO_4_, pH7.4) then spiking with *E. coli* K12 cells. The DNAzyme solution was prepared at a concentration of 500 nM in 2 × RxN buffer containing 2 mg ml^−1^ lysozyme. The bacteria-spiked 20% blood samples and DNAzyme solution were injected via respective inlets at flow rates ranging from 0.5 to 3 μl min^−1^, whereas the oil phase was injected at flow rates ranging from 1 to 5 μl min^−1^. Uniform picolitre-sized droplets were generated at a rate of approximately 2,000 Hz by flow focusing the resulting continuous (oil) phase. To optimize the DNAzyme reaction, different-sized droplets (from 5 to 100 μm in diameter) were generated by tuning the microfluidic channel size and flow rate. A 2-mm magnetic stir bar was placed inside a syringe and was gently rotated by a portable magnetic stirrer (Utah Biodiesel Supply) to prevent precipitation of the blood cells. High-throughput blood encapsulation was achieved by the two-layer droplet microfluidic device with an eight-channel droplet generation structure (flow focusing) on the bottom layer and three inlets on the top layer (two for blood sample and one for DNAzyme solution). The 20% blood samples were injected through the two inlets at a flow rate of 10 μl ml^−1^ then split into eight flow streams. Each blood stream was mixed with 500 nM DNAzyme in 2 × RxN buffer containing 2 mg ml^−1^ lysozyme that was injected through the other inlet. This aqueous mixture was then passed down to the bottom layer through the interlayer connecting hole to the eight-channel flow focusing structure into the oil phase (injected from the bottom layer).

### The 3D particle counter

A 3D particle-counting prototype instrument was built to our specifications (ISS Inc), comprised of a two-channel setup to allow simultaneous red and green fluorescence detection for the rapid quantification of the total number of fluorescent particles in a large volume sample (Supplementary Fig. 6). The apparatus consists of a small microscope that has a horizontal geometry and a mechanical sleeve that holds a cylindrical cuvette of diameter 1 cm. Two motors provide rotational and vertical motion of the cuvette. The software allows the rotational speed to be varied in the 10–1,100 r.p.m. range and the vertical speed in the 1–15 mm s^−1^ range. The vertical and rotational motions are produced, respectively, by the Linear Actuator series (Haydon 3500, Haydon Kerk) and a VEXTA stepping motor model PK233PB (Oriental Motor USA Corp.). These motors are connected to a stage holding the transparent cuvette containing the sample. The excitation light generated by lasers is focused at the volume of observation. The excitation focus is positioned inside the cuvette and relatively close to the wall of the cuvette, at a distance of about 1 mm from the wall. This distance can be adjusted so that detection of particles and analysis could be done even in highly scattering media.

The excitation sources are two diode lasers emitting at 469 nm (ISS Inc) or at 532 nm (Aquaplan). Thus, a particle fluoresces when in the volume of observation. The use of a confocal microscope in combination with simple mechanical motions of the sample container in front of the objective provides the means to move and analyse a sample containing particles through an observation region without requiring a complex optical system comprised of moveable optical components, such as translating optical sources, mirrors or photodetectors. The excitation light from the two lasers are combined in one path through a set of dichroic filters ZT532nbdc and Z470rdc (Chroma Technology Corporation) and directed through a 20 × 0.4 numerical aperture air objective (Newport) to the same volume of excitation.

Fluorescence emitted from the sample is collected by the same objective, transmitted through the set of dichroic filters, focused by a lens into a large pinhole (diameter =2 mm), and then collimated by a second lens to the detectors. A dichroic beam splitter T550lpxr-25mmNR (Chroma Technology Corporation) separates the emission beam into two light paths before its detection by two PMTs (Hamamatsu, HC120-08). Two emission filters (FF01-HQ 500/24- 25 and LP5600; Semrock) are located in front of each PMT. The signal from the PMT is sent to the analog to digital converter and to the acquisition card (IOTech). The sampling frequency is set to 100,000 Hz, corresponding to a time resolution of 10 μs. We systematically optimized the performance of 3D particle counter including various ranges of PMT (200–800), RPM (200–1,000), droplet size (10, 25 and 50 μm) and scanning time (1–10 min).

### Bacterial detection in blood using 3D particle counter and data processing

Droplets containing bacteria in the blood sample, DNAzyme sensor and lysozyme were collected in a cuvette following droplet generation. A 2-ml collected droplet solution contains bacteria at various numbers ranging from 2 to 200,000 cells (corresponding to concentrations of 1–100,000 cells per ml). Specifically, different cell numbers of *E. coli* K12 or clinical isolates were obtained through serial dilution or, at extremely low numbers (1–50 CFU), through a microinjection system (TransferMan NK 2, Eppendorf) were spiked in 100 μl of 20% fresh donor blood. For microinjection experiments, specifically, cultured *E. coli* K12 were diluted with fresh and cold LB (to reduce their mobility) from 10^7^ to 10^9^ times to be easily isolated as single cells in the Luria Broth (LB). To collect a bacterium, a glass microcapillary was loaded in a manual microinjector (CellTram vario, Eppendorf) and the tip of the capillary was adjusted to the position of the target cell under a microscope (Zeiss, Axio Observer A1) using a TransferMan NK2. Single *E. coli* K12 cells were sucked out one by one manually by moving the xyz stage and subsequently were added into blood. Bacteria-spiked blood sample was then microencapsulated with 250 nM DNAzyme using the microfluidic device. In a typical experiment, 100 μl of generated droplets were then mixed with 1.9 ml of pre-made 10% blood droplets (with DNAzyme sensors but not bacteria) in a cuvette and incubated for various time at room temperature. Following the incubation, 2 ml of droplets were analysed for 3–10 min using 3D particle counter at a rotating speed of 400 r.p.m. while translating vertically at a speed of 10 mm s^−1^. Note that that the time of the measurement can be expanded to decrease the errors if necessary^20^,^21^. Collected intensity profiles were analysed with a pattern-recognition filter by SimFCS software (Laboratory for Fluorescence Dynamics). The pattern-recognition algorithm matches amplitude and shape features in the temporal profile to a predetermined pattern that is characteristic of the time-dependent fluorescence intensity of particles passing through the observation volume (see below). The number of detected fluorescent droplets containing bacterium in actual samples was converted into a value of concentration using the calibration factor obtained previously with known numbers of fluorescent bacterium-containing droplets.

### Principle of the pattern recognition algorithm and quantitative criterion for detection

In our 3D particle counting system, the detection of a ‘hit’ is based on a pattern-recognition algorithm (details can be found in our previous publications^20^,^21^). Briefly, a fluorescent particle (droplet in our paper) is detected by the ‘shape’ produced by the passage of the particle in the volume of illumination, which is Gaussian for our instrument. The pattern recognition detects the time of the passage of the particle and the amplitude of the detected pattern. At the same time, the software measures the noise when a particle is absent or non-fluorescent. The hit is considered positive when the residues of the fit of the shape of the particle is above 3 standard deviation (STD) with respect to the noise. Predetermined using fluorescent droplets that contain cleaved or reacted DNAzyme sensors (the ‘standard’), our pattern-recognition algorithm can therefore automatically filter the noise and only report true bacterium-containing, fluorescent droplets.

## Author contributions

D.-K.K., M.M.A., M.A.D., E.G. and W.Z. are responsible for the study concept and design. D.-K.K., M.M.A. and K.Z. carried out the experiments and performed data analysis. D.-K.K., M.M.A. and W.Z. prepared the manuscript. S.H. and E.P. provided clinical input and clinical samples. The content is solely the responsibility of the authors and does not necessarily represent the official views of the NIH.

## Additional information

**How to cite this article:** Kang, D.-K. *et al.* Rapid detection of single bacteria in unprocessed blood using Integrated Comprehensive Droplet Digital Detection. *Nat. Commun.* 5:5427 doi: 10.1038/ncomms6427 (2014).

## Supplementary Material

Supplementary Information Supplementary Figures 1-7, Supplementary Tables 1-3 and Supplementary Methods

Supplementary Movie 1 Blood droplet generation

## Figures and Tables

**Table 1:** Major specifications of the IC 3D test in comparison with a FDA-approved PCR test for bacterium detection.

**Specifications**	**The IC 3D system**	**Typical PCR assay^41^ (for example, FilmArray, BioFire Diagnostics, Inc.)**
Specimen types	Diluted blood	Positive blood culture, sample processing including cell lysis, DNA isolation, PCR, etc
Sample volume	Microlitres to millilitres	Microlitres
Culture enrichment	No	Yes
Amplification	No	Yes
Time to results	≪90 min, yes or no; ≪4 h, quantitative	≪1 h assay+~14 h culture
Limit of detection (CFU ml^−1^)	1–10; Single-cell sensitivity	At the time of blood culture positivity before the test: ~10^7^–10^8^
Selective	Yes	Yes
Quantitative	Yes	No
Linearity	1–100,000 CFU ml^−1^	No
Multiplexable	Yes	Yes

CFU, colony-forming unit; FDA, Food and Drug Administration; IC 3D, Integrated Comprehensive Droplet Digital Detection.
