# Supplementary material for: Rapid detection of single bacteria in unprocessed blood using Integrated Comprehensive Droplet Digital Detection
Source: Nat Commun. 2014 Nov 13;5:5427. doi: 10.1038/ncomms6427 (PMC4243214; doi:10.1038/ncomms6427)
Supplement: Supplementary Information — Supplementary Figures 1-7, Supplementary Tables 1-3 and Supplementary Methods [file ncomms6427-s1.pdf]

Supplementary figures

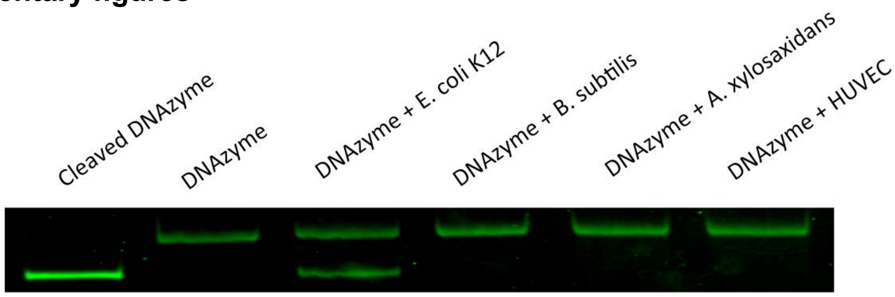

Supplementary Figure 1. Gel electrophoresis data demonstrate that DNAzyme cleaves its fluorogenic substrate only in the presence of target *E. coli*.

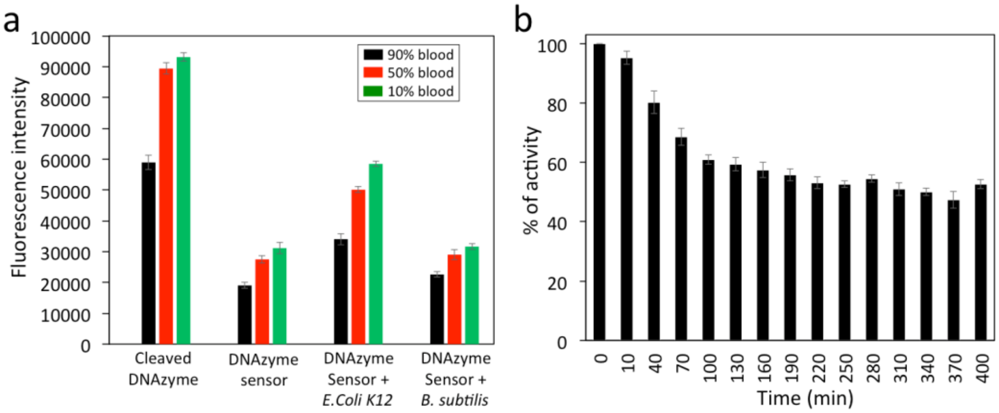

Supplementary Figure 2. **DNAzyme sensors are functional and stable in diluted blood.** a) DNAzyme sensors detect target *E. coli* K12 in the bulk assay in blood diluted by sensor solution at volume ratios of 9:1, 1:1 and 1:9 corresponding to a final blood concentration of 90%, 50% and 10%, respectively. The final solution is 100 μL containing 1000 bacteria, 100 nM DNAzyme sensor and 1 mg ml^{-1} lysozyme. The assay time is 30 min and the reaction was monitored by a fluorescence plate reader. Cleaved DNAzyme sensors (by NaOH/heat) (first set of columns) and intact DNAzyme sensors (second set of columns) were included as positive and negative controls. Our DNAzyme sensors produced measurable fluorescence signal in the presence of *E. coli* in all tested blood concentrations. Data are represented as mean ± s.d., n = 3. b) The activity of *E. coli* DNAzyme sensor incubated in 30% blood at various time before adding bacteria lysates. Data are represented as mean ± s.d., n = 3.

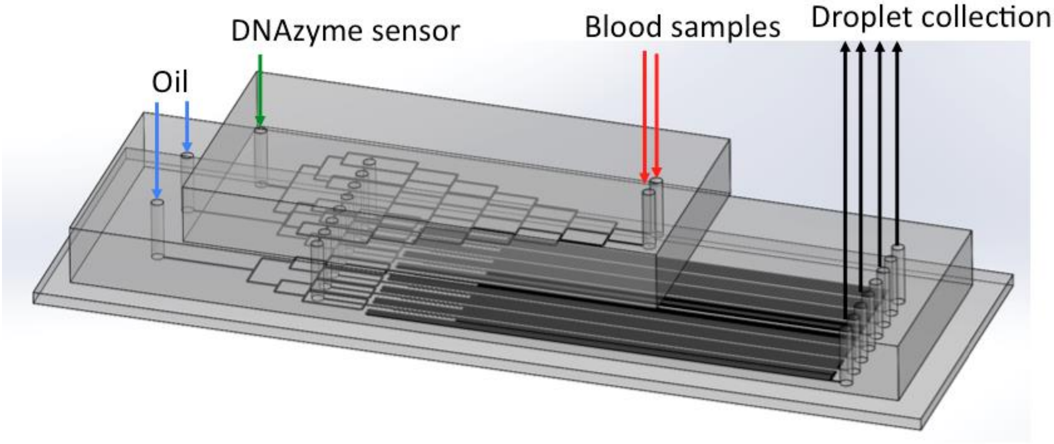

Supplementary Figure 3. **High throughput droplet generator.** A two layer droplet microfluidic device was designed to increase the throughput of droplet generation. Blood samples were introduced through two inlets on the top layer and split into 8 flow streams. The DNAzyme solution was injected through an inlet located on the other side of the top layer and also split into 8 flow streams. The blood and DNAzyme solution streams were merged one-by-one and passed down through the interlayer connecting holes. Mixtures were then encapsulated within 25 μm droplets by the oil phase introduced from the bottom layer inlets.

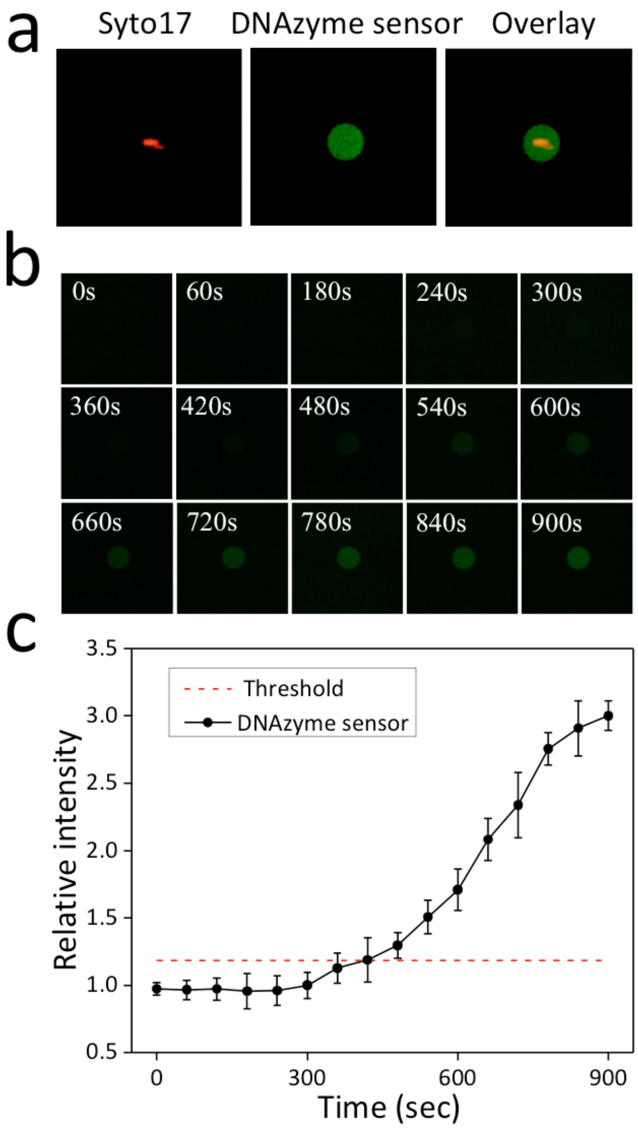
a
Syto17
DNAzyme sensor
Overlay
b
0s
60s
180s
240s
300s
360s
420s
480s
540s
600s
660s
720s
780s
840s
900s
c
Threshold
DNAzyme sensor
Relative intensity
Time (sec)
3.5
3.0
2.5
2.0
1.5
1.0
0.5
0
300
600
900

Supplementary Figure 4. DNAzyme sensors rapidly detect target bacteria *E. coli* in HEPES buffer in droplets. a) Representative fluorescence images show the co-localization of a single Syto17 stained bacterium and DNAzyme sensor signal after 900 s incubation time in the droplet. b) Real-time fluorescence monitoring of a single droplet that contains DNAzyme sensors and a single bacterium. c) Quantification of fluorescence intensity of droplets in experiments described in b). Data are represented as mean ± s.d., n = 3. Threshold was obtained by 'Three sigma rule (three times s.d. of the baseline (relative intensity was stably 1)'. Fluorescent intensity goes above threshold at 8 min. 5 μm droplets are used in this Figure.

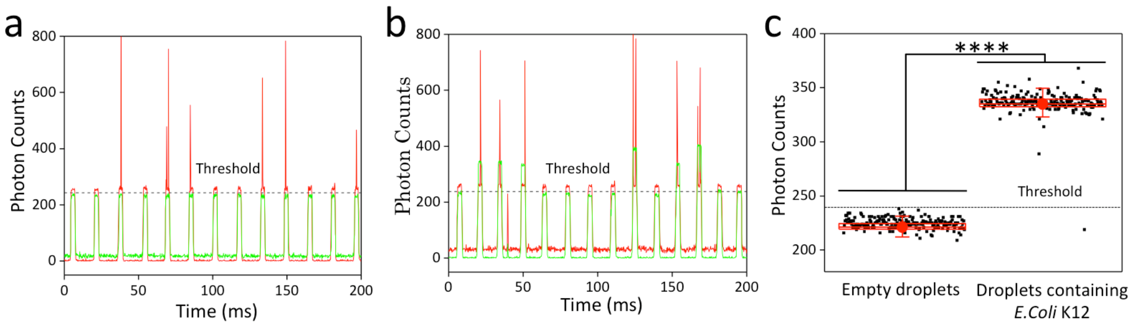

Supplementary Figure 5. SYTO 17 (red color) stained control *Bacillus* (a) or target *E. coli* K12 (b) were spiked in blood at a concentration of 10^7 cells ml^{-1} which was encapsulated in a single cell manner in droplets with DNAzyme sensor (final blood content is 10% in this data). After a 3-hour reaction, droplets are counted on-chip using our confocal detection system. Red spikes represent droplets that contain SYTO 17 stained cells which are observed on both control (a) and target (b) cells. However, only the target *E. coli* K12 (b) produced a green color DNAzyme signal that is above the background (i.e., droplets that do not contain cells). At such a high initial cell concentration (10^7 cells ml^{-1}), there are occasionally 2 bacteria (i.e., 2 red spikes) observed in one droplet. In these cases, the DNAzyme signal directly correlates to the number of bacteria in the droplet. a) and b) were performed in triplicate and a total of ~70,000 droplets were counted. c) Maximum photon counts of representative droplets that contain 0 or 1 *E. coli*. Black dot represents the photon count from each droplet. Box plot with an overlay of actual data is shown. Mean value is shown as red dot. n = 200, ****$P < 0.0001$, Two-tailed Student's *t*-test. A count is considered as a "positive hit" if it is higher than the threshold (dash lines) that is set to be the maximum photon count of empty droplets. This set of experiments reveals that our encapsulated DNAzyme sensor system possesses zero false positive rate and minimal false negative rate (~0.5%) using the 1D on-chip droplet counting.

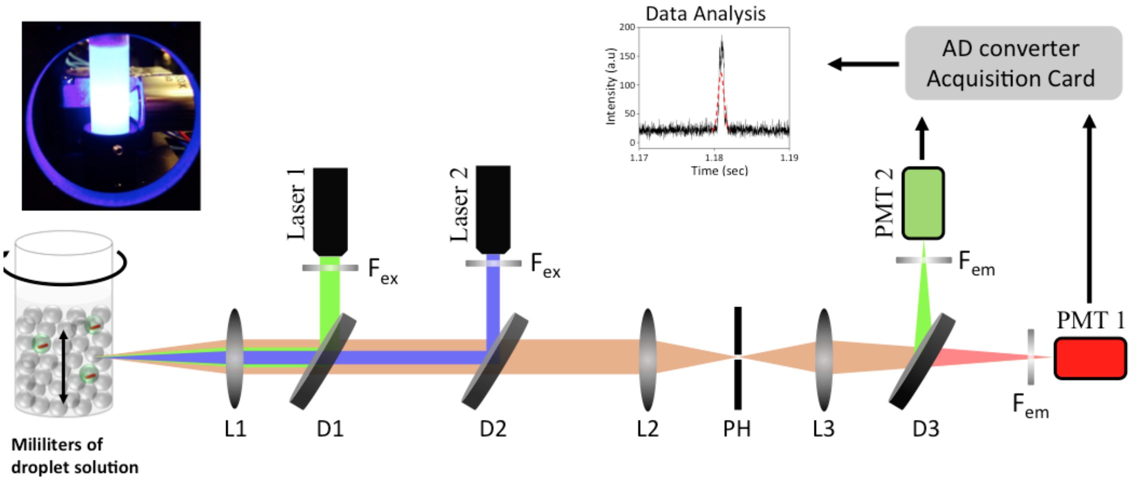

Supplementary Figure 6. **Schematic diagram of 3D particle counter.**[4] Excitation beam from the diode lasers (Laser1 and Laser2) are guided through the excitation filter (F_{ex}) and the dichroic mirrors (D1 and D2). The beam is then focused into the cuvette through the objective lens (L1) (see photo inset). Emission spectra are collected from the same objective lens and transmitted through the dichroic filters, which is focused via a lens (L2) into the pinhole (PH). The light beam is further collimated by another lens (L3) toward the detection unit. A dichroic filter (D3) splits the emission beam before reaching the emission filters (F_{em}) placed in front of the two photomultiplier tubes (PMT1 and PMT2). The analog signals from the PMTs are converted through AD converter acquisition card for data analysis.

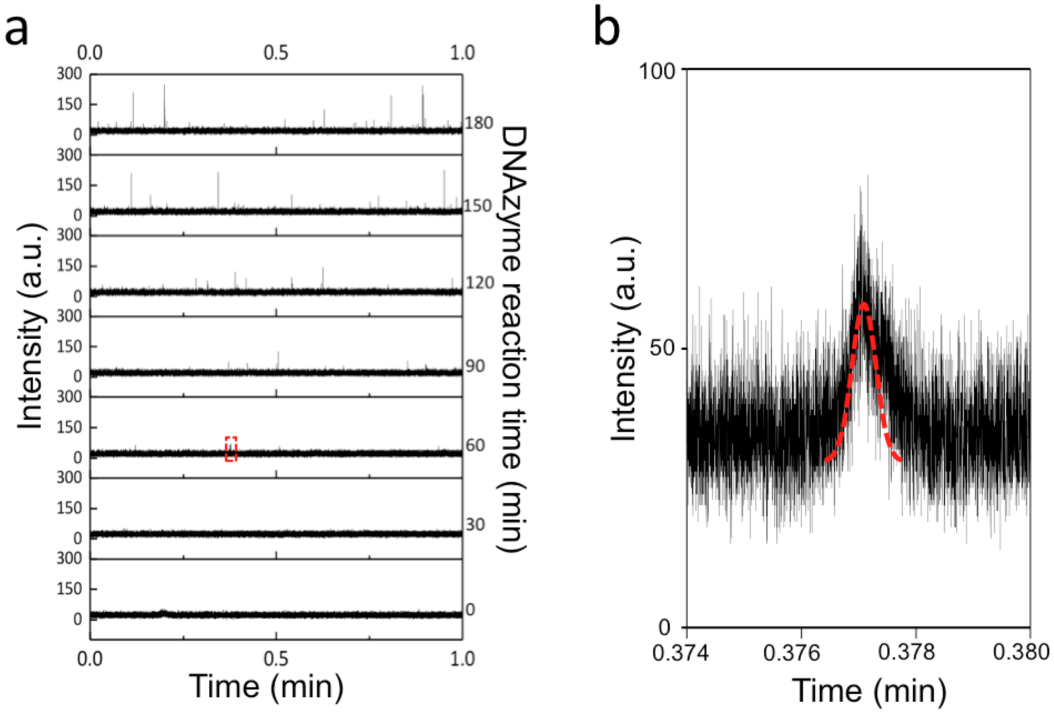

Supplementary Figure 7. **Fluorescent signal kinetics of the DNAzyme reaction in bacterium-containing blood droplets at different reaction time.** a) Time course of fluorescence signal in droplets was monitored every 15 minutes using a 3D particle counter (only some representative time points were shown here). Fluorescence signal of droplets containing single bacterium starts to rise and become measureable at 60 min DNAzyme reaction time. A representative positive signal (red box) is zoomed in and shown in b) where it is analyzed with a pattern recognition algorithm (red dashed line).

Time (min)	1st experiment	2nd experiment	3rd experiment	Number of detected (mean, n=3)	S.D.
0	0	0	0	0.00	0.00
15	0	0	0	0.00	0.00
30	1	1	1	1.00	0.00
45	2	6	3	3.67	2.08
60	23	32	24	26.33	4.93
75	42	53	41	45.33	6.66
90	87	113	90	96.67	14.22
105	114	150	121	128.33	19.09
120	180	224	191	198.33	22.90
135	311	356	302	323.00	28.93
150	490	576	503	523.00	46.36
165	665	760	630	685.00	67.27
180	799	881	801	827.00	46.78
195	869	927	894	896.67	29.09
210	896	954	918	922.67	29.28
225	903	955	906	921.33	29.19
240	913	953	910	925.33	24.01

Supplementary Table 1. Quantification of fluorescent droplets using the 3D particle counter over different DNAzyme reaction time. This table represents the raw data of Fig. 4c and Supplementary Fig. 7.

Sample	Number of spiked	Number of detected (mean)	Error (s.d.)	n
Non-*E. coli* clinical control isolates	N/A*	0	0	8
E. coli K12	0	0	0	5
	1	0.77	0.44	3
	10	7.04	1.75	3
	100	85.93	7.47	3
	1000	894.44	15.78	3
	10000	9505.93	76.27	3

Supplementary Table 2. Quantification of *E. coli* K12 and control clinical isolates from a milliliter of blood droplets using the 3D particle counter. The *E. coli* K12 part of the table represents the raw data of Fig. 4d. *These data are obtained in single-blind test with positive blood cultures of non-*E. coli* isolates including *P. mirabilis*, *M. morganii*, *C. freundii*, *K. pneumonia*, *C. koseri*, *K. oxytoca* and *B. subtilis* (see Fig. 5) although we did not know the cell concentration.

**Components**	**Details (Catalog number, Company, Country) Link for website**	**Price**
650 nm diode Laser	<100mW (FU65011L100-BD22, FU, China) http://www.alibaba.com/product-detail/FU65011L100-BD22-Red-Line-Laser-Module_591784286.html	$36.00
Filters	Filter set for Cy5 including excitation filter, emission filter and dicroic mirror (FF-15, BINGXIN, China) http://www.alibaba.com/product-detail/In-Fluorescence-Microscope-Band-Pass-and_1637192322.html	$150.00
Lens	20× 0.4 NA air objective (LSWJ1-100, Liansheng optics, China) http://www.thorlabs.us/newgrouppage9.cfm?objectgroup_id=7537	$50.00
High-Speed photodetector	400 - 1100 nm, 18 mW, 2 GHz (DET025A, Thorlabs Inc Newton, New Jersey, USA) http://www.thorlabs.us/newgrouppage9.cfm?objectgroup_id=7537	< $200.00
Data Acquisition USB DAQ Device	12-bit resolution, 100 kS/s rate (USB-201, Measurement Computing Corporation, Norton, MA, USA) http://www.mccdaq.com/usb-data-acquisition/USB-201.aspx	< $100.00

Bluetooth module	Mini bluetooth module build-in iBeacon (MDCS71, Nova, China) http://www.alibaba.com/product-detail/new-2014-bluetooth-mini-MDCS71-bluetooth_781447464.html	$10.00
Stepper motor to control the cuvette rotation	2.8 amps/phase, 1.8 degrees / full step (200 steps/revolution) (STP-MTR-23079, Automation Direct, Cumming, GA, USA) http://www.automationdirect.com/adc/Shopping/Catalog/Motion_Control/Stepper_Systems/Stepper_Motors_-z-_Cables/STP-MTR-23079	$45.00
Stepper motor to drive vertical translation	1.8° Stepper Motor and Driver Packages (CVK Series, Oriental Motor USA Corp, Torrence, CA, USA) http://www.orientalmotor.com/products/stepper-motors/CVK2-series-stepper-motors-pulse-input-driver-dc.html	$150.00
Packaging	Case/Optical breadboard platform and packaging all the components	$200.00
Software	iOS or android based software to control 3D particle counter and to analyze number of bacteria using smartphones or tablets	$10.00
Cartridge for microencapsulation	Glass or PDMS based microfluidic device per test	$10.00
Sensor	DNAzyme based bacterium sensor per test	$10.00
Total		< $ 971.00

Supplementary Table 3. Estimated cost break-down of the 3D particle counter.

Supplementary Methods

Bacterial cells and cell culture

E. coli K12 was a kind gift from Dr. Manuela Raffatellu (UC Irvine). Other non-pathogenic bacterial cells, including *Bacillus subtilis* (B. subtilis, ATCC® 82™, ATCC, Manassas, VA, USA), *Pseudomonas peli* (P. peli, LMG 23201, Global catalogue of microorganisms, China), *Achromobacter xylosoxidans* (A. xylosoxidans, ATCC® 212™, ATCC), *Lactobacillus plantarum* (ATCC® PTA-8320™, ATCC), *Pediococcus acidilactici* (P. acidilactici ATCC® PTA-8069™, ATCC), *Amycolatopsis orientalis* (A. orientalis, ATCC® 19795™, ATCC), were purchased. De-identified clinical bacterial isolates were obtained from Dr. Ellena Peterson through the UC Irvine Pathology Research Biorepository. A single colony, freshly grown on an LB agar plate, was inoculated into 5 ml of Luria Broth (LB) and incubated with shaking at 37 °C for 14 h. Ten (10) µL of this culture was further inoculated in 5 ml fresh LB for 4 h then 1 ml LB was transferred to a microcentrifuge tube. The mixture was then centrifuged at 10,000 rpm for 4 min at RT and decanted. After resuspension of the cell pellet in 500 µL of DNAzyme reaction buffer (RxN buffer, 50 mM HEPES (pH 7.5), 150 mM NaCl, 15 mM $MaCl_2$, 0.01 % (v/v) Tween 20), the mixture was heated at 90 °C for 5 min to break the cell wall. Cell lysates were then centrifuged and the supernatant was collected as a sample.

Colony forming unit (CFU) assay

The concentration of bacterial cells was determined by the CFU assay using freshly cultured bacterial cells. *E. coli* K12 or clinical isolates were freshly cultured as described above. Two ml of cultured cells were then serially diluted in 10-fold intervals with LB and divided into two groups; one for stock with 20% glycerol and the other for the CFU assay. Cell concentrations ranging from the 4th to the 9th dilution were then plated onto the agar-LB plates using 100 µL of diluted cells. The bacterial suspension was then

spread evenly over the entire surface of the plate. After drying, the plates were taped to prevent contamination and incubated at 37 °C overnight or until colonies formed. After the colonies reached a suitable size, they were counted to determine the cell concentration.

DNAzyme activity assay in bulk

The fluorescence-based analysis of the cleavage reaction of the construct, as shown in Fig. 2B of the main text, was performed using a fluorescence microplate reader (Synergy HT, Biotek, Winooski, VT, USA) with an excitation wavelength of 488 nm and emission wavelength of 520 nm. To initiate the reaction in the microplate reader, 10 μl of bacterial lysate and 25 μl of 2× RxN buffer were mixed in the microwell plate and 14 μl of H_2O was added to adjust the total volume up to 50 μl. Microwell plates were placed into a microplate reader and the fluorescence intensity was monitored at 1 min intervals continuously following the addition of 1 μl of 2.5 μM DNAzyme or mutant DNAzyme into each well. The fluorescence intensity was recorded for 60 min. All the experiments were performed in triplicate.

Specificity test with different bacterial cells or mammalian cells in bulk

Denaturing polyacrylamide gel electrophoresis (dPAGE)-based specificity assays, as shown in Fig. 2C of the main text, were performed on various cell lysates. For the reaction, 25 μl of 2× RxN buffer was mixed into the microcentrifuge tubes and 14 μl H_2O was then added to adjust the total volume to 50 μl. Following gentle mixing, 1 μl of 2.5 μM DNAzyme was added and mixtures were incubated at room temperature for 30 min. After ethanol precipitation, the DNA molecules were analyzed by 10% dPAGE. The fluorescent DNA bands were visualized by a fluorimager (Typhoon 9200, GE Healthcare, Buckinghamshire, UK). All the experiments were performed in triplicate.

Monitoring DNAzyme sensor signals with clinical E. coli isolates in blood in bulk

Eleven bacterial strains (see above) and fresh blood was obtained from UC Irvine Institute for Clinical and Translational Science (ICTS) through the blood donor program (IRB # 2012-9023). Bacterial cells were freshly cultured in LB and limiting dilutions were performed to quantify the cell concentration as described above. 1,000 CFU of each bacterial strain were then spiked in 25 μl of 20% blood and mixed with 24 μl of 2× RxN buffer. After gentle mixing, 1 μl of 5 μM DNAzyme was added to the mixture before incubating for 30 min at room temperature. The fluorescence intensity was quantified using a fluorescence microplate reader.

Bacterial lysis

Note that lysozyme (1 mg ml^{-1}), a bacteria lysis agent, is pre-mixed in the DNAzyme sensor solution. The use of lysis agents allows the target molecules to be rapidly released from bacteria, which will further decrease the assay time. We have systemically optimized bacteria lysis conditions using various agents including Triton X-100, IGEPAL, SDS and lysozyme alone or in combination, and identified that lysozyme most efficiently lyses bacteria without interfering with droplet formation or DNAzyme sensor function.

1D on-chip detection system

For real-time droplet monitoring on-chip, the fabricated devices were placed onto a translation stage and appropriately aligned with a custom-built confocal spectrometer. The spectrometer excitation source consisted of a 488-nm CW air-cooled argon ion laser. A dichroic mirror (505DRLP02; Omega Optical, Brattleboro, VT) was used to reflect the 488-nm radiation and to define a vertical axis normal to the surface of the optical table. A 40X microscope objective was employed to bring the light to a tight focus

within the microfluidic channel. Subsequently, fluorescence emission was collected with the same high-NA objective and transmitted through the same dichroic mirror. An emission filter (515EFLP; Omega Optical) removed any residual excitation light, and a planoconvex lens (+50.2F; Newport Ltd.) focused the fluorescence onto a precision pinhole (200 μm; MellesGriot, Huntingdon, Cambridgeshire, UK). Another long-pass dichroic mirror (630 LP) was then used to split the signal onto two avalanche photodiodes (AQR-141, EG&G, Perkin-Elmer). Dual detection was implemented for simultaneous recording of green and red fluorescence. Both detectors were coupled to a multifunction DAQ device for data logging (National Instruments, USB-6251) with sub-microsecond time resolution per channel.
